# Nutrition and Diet Patterns as Key Modulators of Metabolic Reprogramming in Melanoma Immunotherapy

**DOI:** 10.3390/jcm14124193

**Published:** 2025-06-12

**Authors:** Katerina Grafanaki, Alexandros Maniatis, Alexandra Anastogianni, Angelina Bania, Efstathia Pasmatzi, Constantinos Stathopoulos

**Affiliations:** 1 Department of Dermatology-Venereology, School of Medicine, University of Patras, 26504 Patras, Greece; efipas@upatras.gr; 2 Department of Biochemistry, School of Medicine, University of Patras, 26504 Patras, Greece; a.maniatis@upnet.gr (A.M.); a.anastogianni@upnet.gr (A.A.); bania.ang@gmail.com (A.B.); cstath@upatras.gr (C.S.)

**Keywords:** nutrition, metabolism, melanoma, immunotherapy

## Abstract

**Background**: Melanoma, one of the most aggressive forms of skin cancer, has seen significant therapeutic advances with immune checkpoint inhibitors (ICIs). However, many patients fail to respond or develop resistance, creating the need for adjunct strategies. **Objective**: The objective of this study is to critically evaluate how specific dietary patterns and nutrient-derived metabolites modulate melanoma metabolism and immunotherapy outcomes, emphasizing translational implications. **Methods**: We performed an integrative review of preclinical and clinical studies investigating dietary interventions in melanoma models and ICI-treated patients. Mechanistic insights were extracted from studies on nutrient transport, immunometabolism, and microbiome–immune interactions, including data from ongoing nutritional clinical trials. **Results**: Diets rich in fermentable fibers, plant polyphenols, and unsaturated lipids, such as Mediterranean and ketogenic diets, seem to contribute to the reprogramming of tumor metabolism and enhance CD8+ T-cell activity. Fasting-mimicking and methionine-restricted diets modulate T-cell fitness and tumor vulnerability via nutrient stress sensors (e.g., UPR, mTOR). High fiber intake correlates with favorable gut microbiota and improved ICI efficacy, while excess protein, methionine, or refined carbohydrates impair immune surveillance via lactate accumulation and immunosuppressive myeloid recruitment. Several dietary molecules act as network-level modulators of host and microbial proteins, with parallels to known drug scaffolds. **Conclusions**: Integrating dietary interventions into melanoma immunotherapy can significantly influence metabolic reprogramming by targeting metabolic vulnerabilities and reshaping the tumor–immune–microbiome axis. When combined with AI-driven nutrient–protein interaction mapping, this approach offers a precision nutrition paradigm that supports both physicians and patients, emerging as a novel layer to enhance and consolidate existing therapeutic strategies.

## 1. Introduction

Melanoma is a highly aggressive form of skin cancer with rising incidence worldwide [1]. Immunotherapy, particularly immune checkpoint inhibitors (ICIs), has revolutionized the treatment landscape for advanced melanoma [2,3,4,5,6,7]. Despite these advancements, a significant proportion of patients either do not respond to therapy or experience disease progression, highlighting the urgent need to explore additional strategies that can enhance treatment efficacy [8].

Diet-based interventions have emerged as a promising field for fine-tuning existing treatments. Nutrition plays a critical role in modulating tumor metabolism and immune function. Personalized dietary interventions, informed by metabolic reprogramming and genetic profiling, can optimize metabolic health and improve immunotherapy response [9,10]. Notably, central metabolic pathways such as glycolysis and glutaminolysis are frequently deregulated in melanoma, leading to rapid proliferation, progression, and survival in the tumor microenvironment (TME). These metabolic demands of malignant melanocytes can be affected and modulated by dietary patterns and specific nutrients with pivotal roles in cancer progression [11]. Moreover, an effective way to fine-tune melanoma metabolism may help overcome resistance to immunotherapy. Preclinical trials suggest that targeting pathways, such as glutamine metabolism, may enhance ICIs’ efficacy [12].

Despite the promising findings, critical gaps in the development of therapeutics specifically designed to modulate melanoma metabolism in clinical settings await further research, clarification, and validation. Integrating precision nutrition into immunotherapy represents a largely unexplored frontier. Few studies have systematically examined how dietary modifications based on individual metabolic and genetic profiles can enhance treatment outcomes [9]. Understanding how diet shapes tumor metabolism and immunity could enable clinicians to incorporate dietary modifications as an adjunct therapeutic tool in melanoma management. The intersection of diet, metabolism, and immunotherapy offers an innovative and largely underexplored area of research that holds promise for improving patient responses and outcomes.

## 2. Materials and Methods

**Figure 1:**
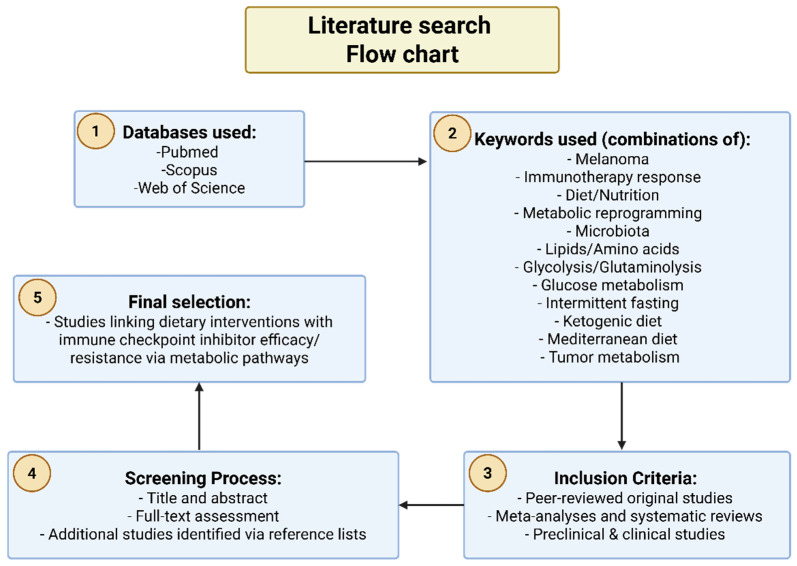
Schematic diagram (flowchart) of the steps involved in the pipeline of the narrative review. All figures were created in BioRender. (https://BioRender.com, accessed on 9 June 2025).

To construct this narrative review, a comprehensive literature search was conducted using PubMed, Scopus, and Web of Science databases for studies published up to April 2025. The primary keywords included combinations of terms such as “melanoma,” “immunotherapy response,” “diet,” “nutrition,” “metabolic reprogramming,” “microbiota,” “lipids,” “amino acids,” “glycolysis,” “glutaminolysis,” “glucose metabolism,” “intermittent fasting,” “ketogenic diet,” “Mediterranean diet,” and “tumor metabolism” (Figure 1) Priority was given to peer-reviewed original research articles, meta-analyses, and systematic reviews. We also included recent preclinical and clinical studies addressing the role of dietary patterns and specific nutrients on immune response modulation and metabolic pathways in melanoma therapy. Articles were screened for relevance based on titles and abstracts, followed by full-text assessment. Manual searches for references from articles were also performed to identify additional relevant publications. The selection process emphasized evidence exploring melanoma mechanisms linking dietary interventions to immune checkpoint inhibitor efficacy and resistance through metabolic pathways.

## 3. Effect of Melanoma Metabolic Heterogeneity on Immunotherapy

### 3.1. Glucose Metabolism and the Warburg Effect

**Figure 2:**
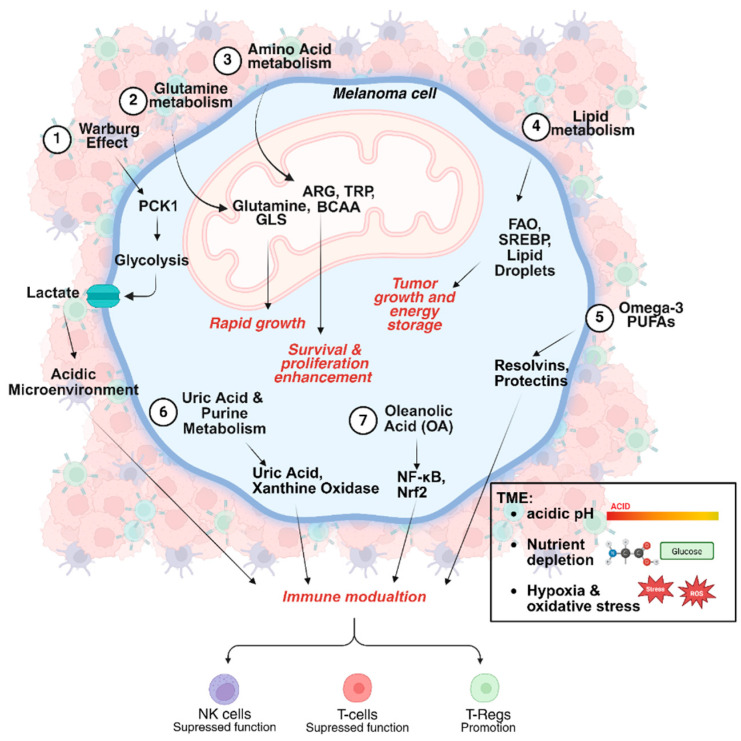
Melanoma cells undergo significant metabolic reprogramming (e.g., glycolysis, glutamine metabolism, PUFAs) to sustain their rapid proliferation and survival. An alteration in immune response is also observed, affecting the tumor microenvironment (TME).

Melanoma cells undergo significant metabolic reprogramming to sustain their rapid proliferation and survival. This metabolic heterogeneity profoundly influences the efficacy of immunotherapy [13]. For example, glucose metabolism in melanoma begins with glycolysis in the cytoplasm, converting glucose into pyruvate, which can then enter the tricarboxylic acid (TCA) cycle and oxidative phosphorylation in the mitochondria to generate energy and essential metabolic intermediates for cancer survival. However, melanoma cells often exhibit dysregulated metabolism, frequently shifting toward aerobic glycolysis (Warburg effect) and producing lactate even in the presence of oxygen [14,15,16]. This shift contributes to the formation of an acidic TME that impairs immune cell function and promotes immune evasion (Figure 2).

Among several important key metabolic regulators, phosphoenolpyruvate carboxykinase 1 (PCK1) plays a central role in reprogramming glucose metabolism in tumor-repopulating cells (TRCs). PCK1, while traditionally associated with gluconeogenesis, exhibits noncanonical functions in melanoma. In B16 TRCs, it promotes the conversion of glucose to lactate, favoring glycolysis over gluconeogenesis. This adaptation supports tumor growth and metabolism, facilitating the ability of TRCs to repopulate tumors. Moreover, PCK1 overexpression contributes to melanoma tumorigenesis and drug resistance, whereas its knockdown inhibits TRCs’ proliferation and tumorigenesis capacity [17]. These findings suggest PCK1 as a potential therapeutic target due to its role in enhancing glycolysis and supporting tumor progression [18,19,20]. In addition, melanoma cells predominantly rely on aerobic glycolysis to generate energy, leading to the accumulation of lactate [21,22]. This metabolic byproduct further acidifies the TME, suppressing anti-tumor immunity by reducing T-cell activation and promoting the recruitment of immunosuppressive cells, including regulatory T-cells (T-regs) and myeloid-derived suppressor cells (MDSCs) [23].

The glycolytic dependency of melanoma restricts essential nutrient availability needed by effector T-cells, weakening their cytotoxic function [24]. Additionally, lactate-induced acidity impairs both cytotoxic T-cells and natural killer (NK) cells while favoring T-reg differentiation, further suppressing the overall immune response [23]. Proton pump inhibitors (PPIs), a common medication, have been demonstrated to neutralize the acidic TME and modify the gut microbiota, thereby impacting the efficacy of ICIs. PPIs in metastatic melanoma patients treated with ICI alone or combined ICIs prolonged survival [25,26,27].

A notable feature of melanoma metabolism is the metabolic symbiosis between cancer cells and stromal cells, which enables metabolic resource sharing within the TME. The solute carrier transporter SLC16A1, involved in lactic acid transport, has emerged as a potential prognostic biomarker mediating immune tolerance. SLC16A1 expression correlates with immune cell infiltration, including CD8+ T-cells and macrophages, suggesting it plays a role in orchestrating immune–metabolic cell dynamics within tumors [28]. Therefore, targeting glycolysis and associated pathways, including lactate transport and amino acid metabolism, can optimize immunotherapy efficacy. Additionally, emerging metabolic biomarkers, such as serum lactate levels and glutamine dependency, may enable personalized precision nutritional and pharmacologic strategies to complement immunotherapy (Table 1) [29,30].

### 3.2. The Role of Mitochondria in Melanoma Survival and Immunotherapy Response

Recent advances, including the introduction of BRAF/MEK inhibitors and immunotherapy, have improved outcomes in advanced melanoma; however, many patients develop resistance, either intrinsically or throughout treatment. This resistance arises through complex mechanisms involving metabolic reprogramming of mitochondria and redox balance, influencing the function of the immune system within the tumor microenvironment [31]. Despite widespread belief in the protective effects of antioxidants against cancer, emerging evidence contradicts this view, particularly in the context of malignant melanoma. Recently, it has been shown that antioxidant supplementation, including compounds like N-acetylcysteine (NAC) and the Vitamin E analog Trolox, can paradoxically promote melanoma metastasis by increasing the migration and invasiveness of human melanoma cells. Mechanistically, this effect was mediated by increased glutathione synthesis and subsequently decreased ROS production, which in turn activated RHOA-signaling, a pathway responsible for cell motility. Conflicting ROS study results reflect the complex role of mitochondria in melanoma and question the universal benefit of antioxidant use in cancer treatment [32].

Another interesting study highlights significant differences in mitochondrial function and immune responses between BRAF V600E-mutated and wildtype melanoma samples through proteomic analysis. In BRAF-mutated tumors, key mitochondrial processes, such as the TCA cycle, oxidative phosphorylation (OXPHOS), and mitochondrial translation, are upregulated, with increased expression of proteins involved in tRNA aminoacylation, rRNA methylation, and Complex I of the OXPHOS system. These alterations support the metabolic reprogramming that drives melanoma progression. Additionally, enhanced RNA polymerase III activity and suppression of immune pathways, like PD-1 signaling, suggest mechanisms for immune evasion. Proteins linked to mitochondrial biogenesis and import, including MTX1/2 and TOMM6, are also elevated, promoting tumor growth and metastasis by altering energy metabolism and redox balance. These findings underscore the complex relationship between mitochondrial activity and immune escape in BRAF-mutated melanoma. Combining mitochondrial inhibitors with BRAF/MEK inhibitors and immune checkpoint blockers offers a promising strategy to improve treatment response and overcome therapeutic resistance [33].

Taken together, the interplay between tumor metabolism, redox state, and TME not only contributes to treatment resistance but may also influence metastatic behavior. This highlights the urgent need for a deeper understanding of the mitochondrial role in metabolic pathways as both therapeutic targets and risk factors in the management of melanoma.

### 3.3. Amino Acid Metabolism and Immune Regulation

Amino acids play crucial roles in tumor metabolism and immune regulation. In melanoma, arginine metabolism is particularly significant. Arginine catabolism by arginase reduces its availability for T-cell function, limiting anti-tumor immunity. Inhibiting arginase enhances CD8+ T-cell activation and persistence, promoting tumor regression [34,35]. Novel approaches combining ICIs with metabolic modulators, such as ranolazine, have shown potential in reversing resistance and improving therapeutic efficacy (Figure 2) [36].

**Figure 3:**
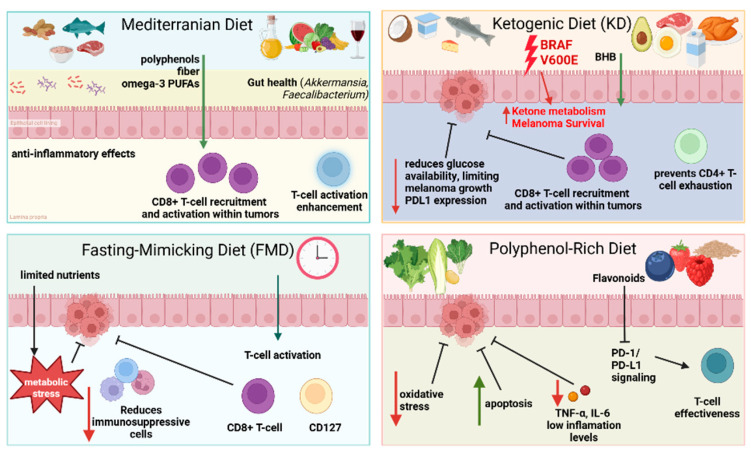
Diet modulates melanoma progression and treatment by influencing metabolic pathways, inflammation, and immune function.

Increased utilization of glutamine provides melanoma cells with the necessary building blocks for their proliferation but also affects immune cell function. While elevated levels of glutamine can enhance the survival and activity of TILs, it simultaneously fuels tumor growth. Oncogenic pathways such as c-Myc drive glutamine metabolism, replenishing critical metabolic intermediates for tumor progression and immune evasion [34]. Targeting glutamine transport can suppress melanoma growth and represents a potential therapeutic target for both BRAF(WT) and BRAF(V600E) melanoma [37]. Additionally, BRAF V600E mutation activates the Oct-HMGCL-acetoacetate axis and MEK–ERK signaling, enabling melanoma cells to use ketone bodies as a metabolic substrate [38]. This mutation-specific metabolic rewiring highlights how ketogenic diets could be detrimental in the context of BRAF ^V600E^ melanoma, emphasizing the need for personalized dietary and therapeutic strategies (Figure 3).

Methionine restriction sensitizes melanoma cells to ICIs by modulating the methionine salvage pathway and methionine adenosyltransferase activity [29,39]. Regulation of the methionine cycle directly impacts T-cell activity, enhancing anticancer immunity [40]. Methionine dependence in melanoma cells underscores a therapeutic vulnerability, suggesting that disrupting this pathway can impair tumor growth and improve therapeutic responses [41]. Furthermore, dietary methionine restriction shifts metabolic balance in melanoma cells, increasing sensitivity to treatment [42].

Asparagine plays a critical role in melanoma immunotherapy by supporting T-cell activation and overall immune function. The metabolism of amino acids, including asparagine, has been shown to influence the activity of anti-tumor CD8+ T-cells, indicating that regulating asparagine levels could enhance the effectiveness of immunotherapy [43,44]. Immune checkpoint inhibitors, such as ipilimumab and nivolumab, work by boosting T-cell-mediated responses against melanoma. Since T-cell proliferation and function depend on nutrient availability, including asparagine, its presence is essential for robust anti-tumor immunity [45,46]. Additionally, biomarkers like PD-L1 expression and tumor mutational burden (TMB) are used to predict responses to immunotherapy [47,48]. Thus, optimizing nutrient access for T-cells, particularly to amino acids like asparagine, may enable more personalized and effective treatment strategies that account for both tumor metabolism and immune system demands [43,44].

Leucine, isoleucine, and valine (collectively termed branched-chain amino acids; BCAAs) have been implicated in melanoma progression. These amino acids are not only vital for protein synthesis and muscle metabolism but also serve as signaling molecules that can activate pathways promoting cell growth and survival [49]. Aberrant BCAA metabolism in melanoma cells promotes lipogenic enzymes, contributing to cancer cell survival and metabolic rewiring [50]. This metabolic reprogramming can create a TME that suppresses immune responses, consequently impacting the outcomes of immunotherapy. Emerging studies have shown that microbial short-chain fatty acids (SCFAs), which are produced from dietary fiber fermentation, can influence CD8+ T-cell responses and improve the efficacy of adoptive immunotherapy [51].

There is increasing interest in combining metabolic inhibitors with immunotherapies to enhance anti-tumor responses. Studies evaluate the effects of combining ICIs with agents targeting metabolic pathways, such as IDO inhibitors that modulate tryptophan metabolism. However, the complexity of metabolic networks suggests that targeting a single pathway may not be sufficient, as compensatory mechanisms can activate alternative pathways, diminishing the therapeutic effects [34]. Therefore, a more comprehensive approach targeting multiple metabolic pathways may be necessary for effective treatment.

### 3.4. Purine Metabolism and the Immunomodulatory Role of Uric Acid

Uric acid, a byproduct of purine metabolism, is implicated in immune regulation through both pro- and anti-inflammatory mechanisms. High serum UA levels have been associated with poor cancer prognosis, including breast and liver cancer [52,53]. In melanoma, the impact of UA on immunotherapy response remains underexplored; however, hyperuricemia has been shown to impair tumor immunity by altering T-cell proliferation and function [54]. Furthermore, a Mendelian randomization study indicated that UA mediates cancer progression through its interaction with fatty acid metabolism [55].

A key determinant of UA’s effects is its dual role in oxidative stress and inflammation. While UA serves as an antioxidant in extracellular environments, intracellular accumulation promotes reactive oxygen species (ROS) production, leading to immune suppression [56]. Recent studies suggest that metabolic reprogramming induced by UA might interfere with dendritic cell activation and antigen presentation, essential for effective melanoma immunotherapy [57,58,59]. Moreover, in the context of melanoma, dietary interventions such as the Mediterranean diet have been proposed to modulate UA levels and improve immunotherapy outcomes [60,61].

### 3.5. Lipid Metabolism and Therapeutic Implications in Melanoma

Fatty Acid Oxidation (FAO) is a critical metabolic pathway in T-cell metabolism and CD8+ function. Enhancing FAO may counteract the immunosuppressive TME and improve T-cell function [62]. Dysregulated lipid metabolism can impair T-cell functions, potentially contributing to immunotherapy resistance. Targeting lipid metabolism could enhance immunotherapy responses, though compensatory metabolic pathways must be considered [34,35]. Metabolic reprogramming of both cancer and immune cells is pivotal in shaping the TME. Melanoma cells often undergo metabolic alterations that support their growth while simultaneously modulating immune cell activity. The metabolic demands of T-cells shift significantly upon activation, since they rely on aerobic glycolysis and FAO, which can be influenced by the metabolic state of the TME [35,63]. This interdependence highlights the need to consider metabolic pathways when designing immunotherapies.

Melanoma cells rely on lipid droplets for energy storage and signaling, highlighting a metabolic vulnerability [64,65]. The lipid droplet-associated protein DHRS3 regulates melanoma cell states, providing insights into metabolic control and tumor progression [66]. Additionally, melanoma cells release extracellular lipidosomes containing lipid droplets and mitochondria during cell division, facilitating novel intercellular communication pathways [67]. Fatty acid-related gene signatures can stratify melanoma prognosis and shape the tumor immune microenvironment (TIME), offering predictive markers for therapy response [28]. Interestingly, combining radiotherapy with immunotherapy promotes lipid oxidation and induces CD8+ T-cell-mediated tumor ferroptosis, a therapy-induced tumor cell death driven by lipid peroxidation [30,68]. The conserved nature of lipid droplets across cell types suggests opportunities for leveraging lipidomics as a biomarker platform in melanoma [69].

Omega-3 PUFAs influence melanoma and skin cancer progression, demonstrating both anti-tumor and immunosuppressive effects. While omega-3 PUFAs reduce inflammation, they may suppress immune responses by enhancing Treg activity, potentially impairing anticancer immunity [70,71]. Therefore, combining omega-3 PUFAs with ICIs may not be advisable. However, specialized lipid mediators like resolvins and protectins effectively reduce tumor-associated inflammation, suggesting that omega-3 PUFAs could enhance anti-tumor effects in skin cancers [72,73,74,75]. Notably, among melanoma patients receiving neoadjuvant checkpoint inhibition, low fiber and omega-3 fatty acid consumption was associated with poor therapeutic response [76]. Finally, oleanolic acid (OA), a triterpenoid found in various plant-based foods, exhibits promising anticancer properties through its immunomodulatory, anti-inflammatory, and anti-proliferative effects [77]. OA influences immune activation by modulating NF-κB and Nrf2 pathways, thereby enhancing cytotoxic T-cell activity and reducing immune evasion by tumors [78].

In melanoma, OA has been investigated for its potential to improve immune checkpoint blockade efficacy by enhancing antigen-presenting cell function and reducing myeloid-derived suppressor cells (MDSCs) that promote immunosuppression [79]. Moreover, OA’s ability to alter lipid metabolism may counteract the metabolic adaptations exploited by melanoma cells to evade immune surveillance [80]. Given its broad spectrum of effects, OA supplementation in dietary interventions could confer a complementary approach to improving immunotherapy response.

## 4. Macronutrients and Their Role in Melanoma Immunotherapy

### 4.1. Protein Intake and T-Cell Activation

A diet low in protein has been shown to reactivate anti-tumor immune response in the preclinical setting [81]. Melanoma mouse models which received an isocaloric diet with protein reduced by 25% compared to controls slowed tumor growth in a CD8+ T-cell-dependent manner, without affecting body weight or blood glucose levels. This effect was attributed to the activation of the unfolded protein response (UPR) by the low-protein diet, which leads to cytokine production and CD8+ T-cell activation following IRE1a (Inositol Requiring Enzyme 1a) and RIG1 signaling [81]. Another small study of 15 patients who participated in metastatic melanoma immunotherapy trials showed significantly higher protein consumption in early versus late responders to immunotherapy, where late response was defined as achieving Complete Remission (CR) after more than nine months since treatment initiation [82].

### 4.2. Sugars and Fructose Metabolism in Immune Evasion

Melanoma cells exhibit a high demand for sugars, such as glucose and fructose, to fuel their rapid proliferation and resistance to apoptosis. The interplay between sugar metabolism and immune modulation is pivotal in TME [83]. Notably, fructose enhances cytoprotection and resistance to immunotherapy in melanoma [84].

In melanoma-bearing mice on a Western diet, those receiving triple immune checkpoint blockade (ICB) targeting PD-1, CTLA-4, and LAG-3 showed diminished response compared to lean controls. This resistance was linked specifically to dietary fructose, independent of adiposity, gut microbiome alterations, or leptin levels [84]. Tumors from models on a fructose-rich diet exhibited increased expression of the 28 kDa isoform of Heme Oxidase (HO-1), a gene regulator of metabolism and oxidative stress responses, also implicated in numerous cancers and therapy resistance [85,86,87,88]. In vitro assays confirmed the involvement of fructose in HO-1 expression and subsequent inhibition of apoptosis [84].

### 4.3. Metabolic Competition and Hyperglycaemia in Immunotherapy Response

Tumor cells utilize sugars not only as an energy source but also to maintain redox balance and drive immunosuppressive mechanisms that impair the efficacy of ICIs. A reciprocal alteration in glucose metabolism exists between melanoma cells and immune cells, mediated by distinct glucose transporters. Melanoma cells preferentially use GLUT1 to increase glucose uptake, while cytotoxic T lymphocytes (CTLs) are deprived of glucose, impairing their function. This metabolic competition significantly affects the success of ICB therapies like anti-PD-1/PD-L1 [83].

Hyperglycemia may further compromise melanoma immunotherapy outcomes via both direct and indirect mechanisms. Metabolically, elevated systemic glucose levels intensify the competitive disadvantage of immune cells within the TME [89,90]. Indirectly, chronic hyperglycemia contributes to systemic inflammation, immune dysregulation, and diminished T-cell function, factors known to impair immunotherapy efficacy [91,92]. In this context, local alterations in TME, combined with systemic metabolic disturbances such as insulin resistance, may synergistically impair anti-tumor immune responses in hyperglycemic individuals [93,94].

Moreover, diabetic patients often exhibit weakened systemic immunity and increased frailty, compounding the metabolic challenges within the TME and potentially affecting clinical outcomes. Notably, hyperglycemia in diabetic patients correlates with reduced survival in advanced cancer patients undergoing immunotherapy, underscoring the detrimental impact of dysregulated systemic glucose levels [95]. ICI-induced diabetes, an autoimmune condition linked to glucose dysregulation, further emphasizes the delicate balance between sugar metabolism and immune responses in patients undergoing ICIs [96]. Interestingly, type 2 diabetes and glucose-lowering medications (GLMs), particularly metformin, have been associated with either neutral or even worsened outcomes in patients undergoing immunotherapy, suggesting that glycemic control alone may not translate into improved therapeutic efficacy [97,98]. Finally, emerging evidence links gut microbiota and response to immunotherapy, suggesting an indirect role of sugar metabolism. Altered gut flora could modulate systemic glucose and energy metabolism, influencing the efficacy of ICIs [99].

The overactivation of oxidative metabolic pathways in melanoma cells exacerbates glucose depletion in the TME, further impairing T-cell function and posing a significant barrier to PD-1 blockade immunotherapy [100]. Interestingly, metabolic adaptability, influenced by FOXP3 expression in CD8+ T-cells, enhances the efficacy of adoptive T-cell therapy [101]. In this context, glucose uptake, as measured by FDG-PET/CT, serves as a biomarker for PFS and immunotherapy response. High glucose uptake often reflects aggressive tumor metabolism and correlates with poorer clinical outcomes [102,103]. Furthermore, hyperpolarized 13C-pyruvate imaging has emerged as a promising tool to assess melanoma response to anti-PD-1 therapy, emphasizing the link between metabolic shifts and therapeutic efficacy [104]. Νon-invasive imaging techniques assessing TME parameters, including metabolic phenotype and plasticity, often change earlier than tumor size due to direct links between oncogenic signaling and metabolic pathways. This is especially relevant in immunotherapy, where TME dynamics influence therapeutic responses, providing insights into treatment monitoring and potential survival outcomes [35,105,106].

Finally, novel therapeutic strategies targeting sugar metabolism are gaining attention. The development of mannose-mimicking glycopolymer nanoparticles offers a promising avenue for enhancing the anti-tumor immune response through DC activation [107]. Similarly, L-fucose modulates DC polarization and function, which is critical for initiating and sustaining an effective immune response against melanoma [108].

### 4.4. Flavonoids and Polyphenols as Potential Adjuvants

Flavonoids have been reported to exhibit pro-apoptotic, anti-angiogenic, and anti-proliferative effects, whereas selenium, despite its antioxidant and radiation-protective properties in preclinical studies [109], has paradoxically been associated with an increased cancer risk in humans. In fact, higher blood selenium has been correlated with a higher cancer incidence. In addition, polyphenols also display anti-angiogenic properties and may reduce melanoma metastasis to the lungs in murine models [110,111,112,113,114].

Flavonoids demonstrate anti-melanoma effects via inhibiting cell proliferation and invasion while inducing apoptosis through mechanisms such as ROS-scavenging, immune modulation, cell cycle regulation, and epigenetic modification, including DNA methylation and histone deacetylation. In the context of melanoma immunotherapy, patients with delayed complete response (more than nine months) consumed higher flavonoid levels than early responders [82]. These compounds target key melanoma-associated pathways (see Figure 2), including p53, Bcl-2, caspases 3 and 9, MAPK, MEK/ERK, PI3K/Akt, and the cyclin-dependent kinase (Cdk) pathways. Flavonoids also inhibit the two-pore channel 2 (TPC2) on the melanosome membrane, enhancing melanin production while reducing melanoma cell proliferation, migration, and invasion, suggesting their therapeutic potential [115]. In addition to direct tumor effects, flavonoids modulate key immune cells like T-lymphocytes, natural killer (NK) cells, and macrophages, enhancing anti-tumor immunity by influencing cytokine production and fostering an immune-permissive tumor microenvironment [116]. Certain flavonoids further inhibit immune checkpoint signaling, including the PD-1/PD-L1 axis, potentially restoring immune surveillance and improving immunotherapy outcomes [116].

Of note, dietary modulation of microRNA (miRNA) expression levels is an emerging field and suggests that nutrition may epigenetically affect gene expression regulation at the transcription level. For example, the polyphenol curcumin has been shown to upregulate miR-205-5p in melanoma models, leading to tumor growth suppression via downregulation of Bcl-2 and PCNA. This miRNA-mediated mechanism may enhance immune responsiveness and represents a novel avenue for diet-based adjunctive strategies [117]. It should also be noted that besides the well-characterized miRNAs, several new small and long non-coding RNAs have emerged as essential modulators of gene expression in melanoma, but their contribution in correlation with nutritional signals remains elusive and calls for further investigation [10,118].

Polyphenolic compounds (e.g., apigenin, luteolin, anthocyanins) exhibit antioxidant, antimicrobial, anti-inflammatory, and anticancer effects, including regulation of PD-L1 expression. Apigenin and its metabolite luteolin suppress IFN-γ-induced PD-L1 in breast and melanoma cells via STAT1 inhibition, further supporting the roles as immunotherapeutic adjuvants [119,120].

### 4.5. The Role of Vitamins and Other Micronutrients in Melanoma Immunotherapy

Vitamins D and C have emerged as key modulators of immune response and tumor behavior in melanoma, particularly in the context of ICI efficacy. Vitamin D supplementation increases the objective response rate and prolonged PFS in advanced melanoma patients undergoing anti-PD-1 therapy, likely via microbiome-dependent mechanisms [121,122]. Additionally, Vitamin D3 downregulates melanoma cell proliferation and migration by regulating *NSUN2* expression [123]. Moreover, Vitamin D may attenuate ICI-related toxicity and adverse events. A retrospective study of patients receiving ICIs revealed that pretreatment intake of Vitamin D (≥400 IU/day) was linked to reduced incidence of colitis, with a dose-dependent effect most pronounced above 1000 IU/day [124]. Vitamin C also contributes to improved immunotherapy outcomes via epigenetic reprogramming. Through alterations in DNA methylation patterns in melanoma cells, Vitamin C increases tumor susceptibility to immune-mediated cytotoxicity, thereby synergizing with ICIs, potentially improving treatment response [125].

While the role of micronutrients in melanoma therapy is promising, their impact on melanoma risk is less clear. Yet, epidemiological data are inconclusive. Caffeine appears protective against UVB-induced carcinogenesis, whereas citrus fruits (e.g., grapefruit) may elevate melanoma risk due to psoralen and furocoumarin content, which sensitize the skin to UVA [126]. Other micronutrients, such as folate, exhibit dual roles, as an anticarcinogenic yet pro-proliferative agent under UV exposure. In contrast, niacin (Vitamin B3), despite its protective role against photosensitivity, has shown no significant association with melanoma risk [127,128,129,130].

## 5. Impact of Dietary Patterns on Melanoma Progression and Response to Immunotherapy

Dietary patterns significantly influence melanoma progression and response to treatments such as ICIs by modulating metabolic pathways, inflammation, immune function, and gut microbiota composition. Although melanoma research has historically focused on genetic and molecular drivers, emerging evidence points to the TME’s susceptibility to dietary modulation through mechanisms such as metabolic reprogramming of tumor cells, affecting ICI outcomes (Figure 3) [131].

Cutaneous melanoma (CM) is genetically heterogeneous and classified into four major subtypes—BRAF-mutant, RAS-mutant, NF1-mutant, and triple wildtype (BRAF/RAS/NF1 wildtype)—with the latter often harboring KIT or GNAQ/GNA11 mutations [132,133]. Oncogenic mutations such as BRAF V600E, NRAS and NF1 not only drive tumorigenesis but also rewire cellular metabolism, including glycolysis and glutaminolysis, thereby shaping the tumor’s immune landscape and responsiveness to ICIs [14,134]. This underscores the importance of integrating genomic profiling with personalized dietary and metabolic interventions.

Moreover, phenotypic plasticity and heterogeneity within melanomas—variations even among cells in the same tumor—are linked to poor prognosis [134]. Subtype-specific metabolic traits further complicate therapeutic responses. Acral melanomas, typically less UV-driven and more genomically stable, exhibit distinct metabolic profiles, including reduced glycolysis and increased reliance on fatty acid metabolism [135,136,137,138,139,140]. These differences may influence how well such tumors respond to ICIs and dietary or metabolic interventions.

Mediterranean dietary patterns rich in plant-based foods, fiber, PUFAs, and antioxidants are linked to improved outcomes in CM patients receiving ICIs. These benefits include enhanced progression-free survival (PFS) and overall response rates (ORRs), likely mediated by anti-inflammatory properties and favorable shifts in the gut microbiome [61]. Furthermore, micronutrients embedded in these dietary patterns, such as Vitamins D and C, may enhance immune-mediated tumor suppression, as previously discussed [119,121,122]. Interestingly, a plant-based diet exhibited a favorable response to anti-PD-1, whereas a diet comprising dairy products had an unfavorable effect [141].

These insights underscore the potential of precision nutrition, where individualized dietary interventions based on tumor metabolic profiles and patient-specific characteristics could optimize melanoma management and immunotherapy efficacy.

### 5.1. Mediterranean Diet and Immunotherapy

The therapeutic relevance of the Mediterranean diet in melanoma is currently under investigation in the MINI-MD Study (NCT06236360), which evaluates personalized, telehealth-based dietary interventions in metastatic melanoma patients undergoing immunotherapy. The trial aims to assess the impact on gut microbiome composition, clinical biomarkers, and quality of life compared to a standard diet.

Supporting this approach, a recent cohort study found that adherence to a Mediterranean dietary pattern is associated with improved outcomes in patients with advanced melanoma receiving ICB therapy. Specifically, the study indicated that patients who followed the Mediterranean diet had a higher probability of PFS and a better ORR to ICB treatment compared to non-adherents to this dietary pattern. The proposed mechanisms include the diet’s antioxidant and anti-inflammatory properties, as well as its ability to promote a more immunostimulatory gut microbiome [61]. As ongoing clinical trials continue to clarify causality, these strategies may pave the way for evidence-based dietary recommendations in melanoma management.

### 5.2. Ketogenic Diets and Tumor Metabolism

Ketogenic diets (KDs) have demonstrated tumor suppressive effects in melanoma models. In immunocompromised mice, KDs reduced tumor growth across metabolically heterogeneous human melanoma xenografts. In immunocompetent mice, KDs reduced metastasis and modulated amino acid metabolism, particularly reducing alpha-aminoadipic acid, a cancer biomarker [142]. In orthotopic melanoma models, a ketogenic diet with a 4:1 fat/protein ratio enhanced CD8+ T-cell activity and reduced tumor size in a T-cell-dependent manner, an effect attributed to elevated 3-hydroxybutyrate. Administration of sucrose reversed the ketogenic metabolic benefits. Intermittent KD schedules synergized with ICIs by preventing CD4+ T-cell exhaustion and reducing the expression of resistance markers such as Lag3 on CD8+ T-cells. Although CTLA4 and PD-1 expression levels persisted, expression of their respective ligands on splenic cells, CD86 and PDL1, was decreased, sustaining T-cell activation [143]. Thus, ketogenic interventions may reshape the TME to favor sustained immune activation without inducing early T-cell exhaustion.

### 5.3. Fasting-Mimicking Diets and T-Cell Activation

Fasting-mimicking diets (FMDs), characterized by short-term caloric restriction followed by a return to normal diet, have gained attention as a potential adjunct to melanoma immunotherapy. FMDs in synergy with ICIs enhance T-cell activity, reduce the population of immunosuppressive myeloid-derived suppressor cells (MDSCs), and promote a nutrient-deprived TME that favors immune activation [144,145]. FMDs, when combined with antibodies targeting PD-L1 and the co-stimulatory molecule OX40 (CD134), further amplify effector T-cell infiltration, suppress regulatory T-cells, and upregulate CD127 expression, thus sustaining anti-tumor activity [146]. Notably, even in the absence of immunotherapy, FMDs alone have demonstrated CD8+ TILs and chemotherapy efficacy in melanoma models [147] (Table 2).

Table 2 summarizes key dietary patterns that influence melanoma immunotherapy outcomes, including their underlying biological mechanisms and clinical effects. To facilitate practical translation into patient care, representative foods for each dietary approach are also included. These examples aim to guide the development of patient-centered nutritional strategies that align with emerging evidence on diet–immunotherapy interactions.

## 6. Obesity-Driven Changes in Melanoma Immunotherapy

**Figure 4:**
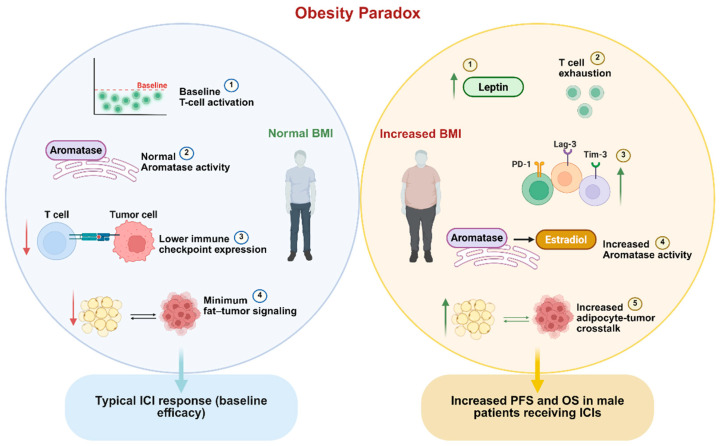
Contrasting immune and metabolic features in lean vs. obese melanoma patients reveals mechanisms underlying the obesity paradox. Despite increased leptin levels and T-cell exhaustion markers (PD-1, Tim-3, Lag-3) in obese patients, especially males, obesity is associated with improved response to immune checkpoint blockade (ICB). Enhanced aromatase activity in adipose tissue increases estradiol levels, potentially modulating immune function. These effects contribute to increased progression-free and overall survival in specific subgroups, highlighting complex immunometabolic dynamics.

It is unambiguously clear that obesity has been linked to cancer via several molecular mechanisms and represents a considerable risk factor [148,149]. However, in a recent meta-analysis, patients with higher Body Mass Index (BMI) experienced favorable outcomes under ICI treatment in melanoma, among other cancers, a phenomenon described as the Obesity Paradox (Figure 4) [150]. More specifically, increased BMI is associated with increased PFS and overall survival (OS) in male melanoma patients receiving the anti-CTLA4 agent ipilimumab plus the chemotherapeutic agent dacarbazine or PD1-PDL1 axis inhibition. The authors attribute this to the interplay of metabolism and signaling pathways related to carcinogenesis, rather than pharmacokinetics, since immunotherapy dosage is calculated based on weight. Interestingly, this effect was not replicated with obese females, since obesity may confer a survival advantage in male patients treated with immunotherapy, potentially due to increased levels of circulating estradiol resulting from adipose tissue aromatase activity [151,152].

Class I obesity (BMI = 25–35 kg/m^2^) and male gender correlated with the longest overall survival and PFS among normal-weight and class II or III obese patient subgroups receiving single or dual ICB [153]. Overweight patients (BMI ≥ 25 kg/m^2^) receiving ipilimumab demonstrated improved response rates compared to patients with normal BMI, but improvements in OS did not reach statistical significance. In this cohort, however, this effect was observed regardless of gender [154]. Conversely, sarcopenia has been linked to poorer outcomes across various cancer types, including melanoma, highlighting the complex relationship between body composition and treatment efficacy. The presence of adipocytes surrounding melanoma tumors has been associated with increased tumor progression, suggesting that metabolic interactions between adipose tissue and tumor cells can adversely affect treatment outcomes [155]. The favorable effect of overweight or obese-level BMI on prolonging time to treatment failure, PFS, and OS has also been demonstrated for patients receiving anti-PD1/anti-PDL1 immunotherapy against melanoma, among other tumors [156].

The apparent favorable effect of obesity on immunotherapy response in melanoma is rather paradoxical, as obesity is linked with a chronic inflammatory state [157]. Obese melanoma mice tumors grow more rapidly and demonstrate leptin-induced T-cell exhaustion via overexpression of PD1, Lag3, and Tim3. However, they paradoxically respond better to PD1 blockade and have reduced metastasis risk [158]. In diet-induced obese mouse models, anti-PD-L1 treatment was effective only in female obese mice, whereas lean mice responded regardless of sex. Strategies like PPARγ inhibition enhanced anti-PD-L1 effects more in lean than obese mice, with no impact in obese males [159].

## 7. Gut Microbiota and Melanoma Immunotherapy

A healthy gut microbiome has been associated with improved responses to immunotherapy in melanoma, as specific microbiota species define distinct patient subgroups with better prognosis [160,161,162]. More specifically, *Faecalibacterium* species (e.g., *Faecalibacterium prausnitzii*) was associated with prolonged PFS, while *Bacteroidales* was associated with a shorter PFS in metastatic melanoma patients treated with anti-PD1 or anti-CTLA4 agents [160,163]. Responders exhibited higher CD8+ T-cell infiltration, enhanced cytokine responses to PD-1 blockade, and a circulating effector T-cell profile, in contrast to non-responders who show a predominance of regulatory and myeloid suppressor cells. These differences may originate from microbiota-driven metabolic programs, particularly anabolic pathways such as amino-acid biosynthesis, which is crucial for immune defences [160].

Although a plethora of studies report variations in microbiome composition between responders and non-responders to ICI, a consistent predictive bacterial signature remains elusive [82]. Nonetheless, treatment response is frequently associated with greater microbial diversity and enrichment of beneficial taxa such as *Akkermansia* and *Bifidobacterium* [164,165,166]. Additionally, intratumoral *Lactobacillus reuteri* also enhances ICI efficacy by producing tryptophan metabolites that promote anti-tumor immunity [167]. These findings suggest that dietary interventions or supplements that improve microbiome composition could potentiate immunotherapy outcomes [168]. In mouse models, supplementation with eight microbiota strains increased gut and blood short-chain fatty acids (propionate and butyrate), promoted Th17 T-cell recruitment through the CCL20/CCR6 axis, and provided Th17-dependent protection against melanoma lung metastasis, in a tissue-specific manner [169]. Probiotic-treated immunocompetent mice showed tumor growth suppression, while prebiotics increased Tumor Infiltrating Lymphocytes (TILs), particularly effector CD4+ and CD8+ T-cells, and activated dendritic cells (DCs). Gene expression analysis revealed upregulated chemokines, antigen presentation, and inflammasome pathways, along with reduced circulating IL-6 and IL-17 [170,171,172].

Modulation of the gut microbiome has been shown to influence both efficacy and toxicity of cancer immunotherapy. In mouse models, CTLA-4 antibody treatment led to increased *Clostridiales* and reduced *Bacteroidales* and *Burkholderiales*, while its efficacy was diminished by broad-spectrum antibiotics. PD-1 blockade was associated with elevated *Bifidobacterium* species in mice showing delayed tumor growth and better therapeutic response. Most importantly, the use of antibiotics compromises the efficacy of both PFS and ICB in melanoma patients [165,173,174,175,176,177,178]. The microbiome’s modulatory role in ICI response can be further improved by its modulation using fecal microbiome transplantations from clinical responders or healthy donors [179,180,181].

### 7.1. Nutrition-Related Clinical Trials

Probiotic use may disrupt gut microbial diversity essential for effective ICI responses, impairing TH1 immunity and antigen presentation. Self-reported probiotic use in clinical settings was not significantly associated with ICB response or survival. However, probiotic administration reduced the efficacy of anti-PDL1 therapy in melanoma mouse models by reducing microbial diversity and dendritic cell function [182]. These findings are further clarified by contrasting murine and limited human data so far [179,182].

Currently, a Phase I clinical trial (NCT05967533) is evaluating the immunomodulatory effects of a standard wheat germ supplement in patients receiving ICIs for solid tumors, including melanoma. In contrast, higher dietary fiber intake was associated with improved ICI response and survival. Notably, the most favorable outcomes were observed in individuals consuming high fiber without probiotics, suggesting that fiber-rich diets may enhance immunotherapy via gut microbiome modulation and expansion of beneficial bacterial taxa [182]. In PD-1-treated melanoma models, high dietary fiber intake led to increased CD4+ T-cell infiltration, upregulation of T-cell activation and interferon response genes, and reduced fecal propionate levels. These effects were absent in germ-free mice, reinforcing the microbiome’s pivotal role in immunotherapy responses [182].

The ongoing Phase II DIET Study (NCT04645680) investigates the impact of dietary fiber on the gut microbiome composition and immune responses in Stage III/IV melanoma patients receiving anti-PD1 therapy. Participants are assigned isocaloric whole-food diets differing in fiber content. Another trial (NCT04866810) examines the combined effect of diet and exercise, comparing standard counseling to a structured intervention including a plant-based, high-fiber diet alongside a prescribed exercise regimen of at least 150 min of moderate or 75 min of high-intensity activity per week. These clinical trials aim to define evidence-based nutritional strategies for enhancing ICI efficacy.

Furthermore, NCT06466434 aims to evaluate the effect of a prebiotic food-enriched diet (PreFED) on the response to ICB and its effect on the abundance of gut microbiota *Faecalibacterium* species. Additionally, NCT05303493 Camu-Camu berry (*Myrciaria dubia)* has prebiotic potential to enrich *Akkermansia muciniphila*, a bacterium shown to alleviate metabolic disorders and improve ICI efficacy in preclinical models, and its safety and tolerability will be assessed.

Collectively, emerging trials and preclinical evidence support integrating dietary modulation and metabolic reprogramming into melanoma immunotherapy paradigms. Advancing these concepts from preclinical findings to clinical application will be essential for achieving more durable and personalized immunotherapy outcomes (Table 3).

### 7.2. Personalized Nutrition Strategies for Melanoma Patients

Emerging evidence suggests that integrating dietary counseling into oncology clinics could play a transformative role in melanoma care. Identifying patients with suboptimal gut microbiota profiles could lead to targeted interventions, such as high-fiber diets or selective prebiotics, which may improve ICI responses [182]. Additionally, metabolic profiling using advanced techniques like mass spectrometry or nuclear magnetic resonance could help stratify patients based on their tumor’s metabolic dependencies, enabling tailored nutritional strategies that complement existing therapies [23]. Despite its promise, precision nutrition faces implementation barriers. Interindividual dietary response variability, challenges in clinical dietary assessment, and limited collaboration among oncologists, dietitians, and researchers hinder integration [183]. However, digital tools, such as mobile apps for dietary tracking and AI-driven analysis of metabolic profiles, offer a practical pathway to overcome these hurdles [184]. Translation into clinical practice must consider adherence challenges, especially for complex diets like ketogenic or fast-mimicking regimens. Treatment-related fatigue, gastrointestinal toxicity, and socioeconomic barriers further complicate compliance. Importantly, malnutrition and cachexia risk in advanced melanoma may render restrictive diets harmful. Thus, nutritional screening and patient-centered interventions are essential.

Patients often seek actionable dietary advice following a cancer diagnosis. They view diet as a modifiable factor in their treatment journey, seeking empowerment through lifestyle modifications [185]. A survey of oncology patients revealed that 83% believed diet has a significant impact on treatment outcomes [186]. However, the lack of clear, evidence-based dietary guidelines for melanoma patients fosters confusion and reliance on misinformation. Clinicians should promote practical, evidence-based strategies, for example, to emphasize the benefits of a Mediterranean diet or high fiber intake, while avoiding unnecessary supplementation that could impair treatment [61,187]. It is also essential to differentiate caloric restriction (CR) from macronutrient-specific interventions in melanoma care. CR involves reduced total energy intake and promotes autophagy and immune surveillance in preclinical models. In contrast, nutrient-targeted approaches, such as protein or methionine restriction, modulate specific metabolic pathways critical for tumor growth and immune regulation without necessarily reducing total caloric intake. Such distinctions point to the alignment of dietary strategies with specific immunologic targets.

Patient educational tools, such as printed materials or webinars on Mediterranean diets, fiber intake, or fasting, can enhance understanding and adherence. Practical guidance, like meal planning tips or affordable sources of probiotics, may further support patients [61,186]. Moreover, engaging patient advocacy groups in the clinical trial design can improve patient-centered approaches and participation. Incorporating patient feedback may also help refine trial endpoints, ensuring they align with real-world expectations, such as quality of life and diet sustainability [186,188].

Ultimately, the development of standardized dietary guidelines, informed by high-quality clinical evidence, could address a critical gap in melanoma care (Table 4). Organizations like the American Cancer Society and ESMO could lead these efforts, ensuring consistent recommendations for both patients and healthcare providers [61,189].

## 8. Metabolic Influence in Melanoma Immunotherapy: Lessons from Other Cancers

Across cancer types, metabolic pathways exhibit both unique and shared characteristics. Although melanoma has distinct metabolic and immune characteristics, similarities exist with other cancers where diet and metabolism play crucial roles. In melanoma, the reliance on aerobic glycolysis parallels findings in other cancers, such as glioblastoma and non-small cell lung cancer [190,191]. However, melanoma’s metabolic landscape uniquely intersects with immune pathways, particularly through lactate production, which impairs T-cell function, a phenomenon less pronounced in colorectal or breast cancer [190,192].

Similarly, ketogenic diets have been studied in breast cancer for their potential to starve tumor cells of glucose, an approach that aligns with the metabolic-targeting strategies in melanoma. These diets have also shown promise in both melanoma and glioblastoma models, possibly through mechanisms involving reduced glucose availability [142,193]. Conversely, high-fiber diets, which modulate the gut microbiome, are particularly impactful in cancers with significant microbiome–tumor interactions, such as melanoma and colorectal cancer [194]. These comparisons underscore the need for cancer-specific dietary strategies that consider the tumor microenvironment and systemic metabolic factors. In colorectal cancer, the gut microbiome significantly influences immunotherapy response, echoing findings in melanoma regarding the role of dietary fiber and probiotics [191,194]. Interestingly, melanoma’s obesity paradox, where increased BMI correlates with better immunotherapy outcomes in some populations, contrasts with findings in breast and pancreatic cancers, where obesity accelerates tumor progression [195]. This discrepancy may stem from differences in adipose tissue signaling and its impact on the tumor–immune interface, highlighting the complexity of developing universal dietary recommendations for cancer patients and the need for tumor-specific dietary strategies tailored to unique metabolic dependencies.

## 9. Limitations and Conclusions

The interplay between diet, metabolism, and immunotherapy in melanoma reflects a complex, multifaceted landscape that both aligns with and diverges from findings in other cancer types. Melanoma exhibits unique metabolic–immune interactions compared to other cancers although it shares certain metabolic features with cancers such as glioblastoma and non-small cell lung cancer, particularly its reliance on aerobic glycolysis. The parallels and distinctions across cancers emphasize the need for cancer-specific dietary strategies that reflect the metabolic characteristics of the tumor and its microenvironment. An intriguing example of tumor-specific metabolic influence is melanoma’s “obesity paradox,” where increased BMI correlates with improved outcomes to immune checkpoint inhibitors, contrary to findings in other cancers. This discrepancy may arise from differences in adipose tissue signaling and its influence on immune activity, further complicating efforts to create generalized dietary guidelines for cancer patients.

Despite promising findings, significant contradictions remain in the literature, especially regarding dietary schemes and the role of several micronutrients for which little is known. Moreover, the lack of creditable information deriving from large cohorts remains an obstacle with the heterogeneity of dietary effects on protein metabolism as an example. This highlights the context-dependent nature of nutrient–immune interactions and calls for deeper mechanistic studies to clarify these relationships and guide dietary recommendations. Existing clinical studies often suffer from methodological limitations, including reliance on self-reported dietary data prone to recall bias, small sample sizes, and a lack of diversity among study participants. These issues constrain the generalizability and reproducibility of findings. Additionally, numerous confounding variables—including baseline nutritional status, physical activity, and concurrent therapies—complicate interpretation and must be systematically addressed in future research.

To move the field forward, future research should prioritize multicenter randomized controlled trials that enroll diverse patient populations and employ standardized dietary and metabolic assessments. Longitudinal study designs are essential to evaluate the durability of dietary effects on immunotherapy response. Integration of advanced technologies—such as metabolomics, transcriptomics, and microbiome sequencing—will enhance mechanistic insight and support the development of targeted, evidence-based dietary strategies tailored to melanoma’s unique immunometabolic context. A major barrier to clinical translation is the gap between tightly controlled preclinical models and the physiological, psychological, and metabolic variability of human patients. Moreover, animal models cannot fully replicate the complexity of human tumor biology, immune system dynamics, and dietary responses, limiting the extrapolation of findings. Anatomical and physiological limitations, distinct gut microbiota, and ketone metabolism project the value of humanized models and patient-derived xenografts in better recapitulating human melanoma metabolism, yet challenges remain and complementary human studies are important [196,197,198].

In conclusion, emerging evidence underscores the potential of dietary interventions to modulate tumor metabolism and enhance the efficacy of immunotherapy in melanoma. Nutritional schemes such as the Mediterranean diet, ketogenic regimens, and intermittent fasting appear to influence immune function, the tumor microenvironment, and systemic metabolic states, which are pivotal in shaping therapeutic responses. While preclinical models offer compelling mechanistic insights, human data remain limited and heterogeneous. Bridging this gap requires rigorously designed clinical trials to validate the therapeutic impact of specific dietary patterns and to integrate them as adjuncts in precision oncology. Understanding the dynamic interplay between diet, host metabolism, gut microbiota, and immune responses holds promise for refining melanoma treatment paradigms and optimizing patient outcomes.

## Figures and Tables

**Table 1:** Summary of key biomarkers implicated in melanoma metabolic pathways and their relevance to immunotherapy efficacy.

Biomarker	Associated Pathway/Function	Role in Melanoma Immunotherapy
PCK1	Glucose metabolism (glycolysis)	Promotes tumor-repopulating cell growth; overexpression linked to drug resistance; potential ICI target
SLC16A1 (MCT1)	Lactate transport	Correlates with immune infiltration (CD8+ T-cells, macrophages); prognostic marker candidate
Serum lactate	Glycolysis/ TME acidification	High levels reflect glycolytic flux, linked to an immunosuppressive microenvironment
Glutamine dependency	Amino acid metabolism	Fuels tumor growth: Transport inhibition enhances ICI efficacy
Arginase	Arginine catabolism	Depletes arginine, impairing T-cell function; inhibition boosts CD8+ T-cell responses
Methionine	Methionine salvage pathway	Restriction reprograms T-cell metabolism; sensitizes tumors to ICIs
Asparagine	T-cell activation	Essential for CD8+ T-cell proliferation and function
BCAAs (Leu, Ile, Val)	Lipid and protein metabolism	Promote immune suppression via metabolic rewiring in the TME
Uric acid (UA)	Purine metabolism	High levels impair T-cell immunity, linked to fatty acid metabolism and poor outcomes
DHRS3	Lipid droplet regulation	Controls melanoma cell phenotype; metabolic vulnerability
HO-1	Fructose metabolism/oxidative stress	Induced by fructose; contributes to immune evasion and therapy resistance
PD-L1 expression	Immune checkpoint signaling	Predictive biomarker for ICI response
Tumor mutational burden (TMB)	Genomic instability	Higher TMB predicts greater ICI responsiveness
CD127	Il-7 receptor on T-cells	Upregulated by fasting-mimicking diets; enhances T-cell survival/function
FDG-PET/CT uptake	Glucose uptake/imaging biomarker	High uptake correlates with poor ICI outcomes
Hyperpolarized 13C-pyruvate	Glycolytic imaging biomarker	Assesses early ICI therapy response in vivo
Microbiota markers	Gut microbiome composition	*Faecalibacterium* linked to improved PFS; *Bacteroidales* to reduced response
IL-6, IL-17	Inflammatory cytokines	Lower levels correlate with improved ICI response and prognosis

**Table 2:** Dietary patterns associated with mechanisms influencing melanoma immunotherapy outcomes and food examples.

Dietary Pattern	Mechanism	Effect on Immunotherapy	Example Foods
Mediterranean Diet	Enhances microbiota, reduces inflammation	Improves PFS and ORR	Olive oil, fish, nuts, legumes, leafy greens
High-Fiber Diet	Supports beneficial gut microbiota, enhances immunity	Enhances ICI efficacy, extends survival	Lentils, oats, beans, and berries
Ketogenic Diet Ketogenic Diet + BRAF^V600E^ mutation	Reduces glucose, increases ketones, and CD8+ T-cell function Activates ketone metabolism via Oct1-HMGCL axis	Reduces tumor growth, synergizes with ICIs May induce ICI Resistance, genotype-dependent caution	Avocado, cheese, nuts, and coconut oil As above: **Not recommended** in BRAF V600E)
Fasting-Mimicking Diet	Metabolic stress reduces immunosuppressive cells	Enhances T-cell infiltration, improves ICI response	Vegetable broths, nuts, and zucchini
Low-Protein Diet	Triggers the UPR pathway and cytokine signaling via IRE1 and RIG1	Promotes CD8+ T-cell activation (preclinical)	Green peas, mushrooms, oats
Methionine Restriction	Alters T-cell and tumor metabolism	Sensitizes tumors to ICI	Spinach, broccoli, and limited red meat
Western/High-Sugar Diet	Promotes lactic acid, increases immunosuppression	Reduces ICI efficacy, increases resistance	**To Avoid:** Soda, pastries, sugary cereals
Flavonoid-Rich Diet	Modulates immune checkpoints, reduces oxidative stress	May enhance anti-tumor immunity	Onions, apples, berries, citrus, green tea

**Table 3:** Clinical trials investigating diet, nutrition, and lifestyle in melanoma immunotherapy.

Trial Name/ID	Focus/Intervention	Population/Setting	Primary Outcomes	Status/Notes
MINI-MD Study NCT06236360	Mediterranean diet via telehealth coaching	Metastatic melanoma on ICIs	Microbiota, QoL, clinical biomarkers	Ongoing, interventional Phase II
DIET Study NCT04645680	High fiber vs. standard	Stage III–IV melanoma on anti-PD1	Microbiome composition, immune activation	Phase II, ongoing
Camu-Camu Berry Trial NCT05303493	Camu-Camu supplement to enrich *Akkermansia muciniphila*	Solid tumors, incl. melanoma	Safety, tolerability, and microbiome enrichment	Phase I, recruiting
PreFED Study NCT06466434	Prebiotic food-enriched diet to increase *Faecalibacterium*	Patients on ICIs	ICB response, microbiota profiling	Early-phase trial, ongoing
Diet + Exercise Trial NCT04866810	High-fiber, plant-based diet + structured exercise	Melanoma and solid tumors on immunotherapy	Immune markers, fitness, body composition, and ICI efficacy	Active lifestyle intervention study
Wheat Germ Supplementation Trial NCT05967533	Standard wheat germ extract supplementation	Solid tumors, incl. melanoma	Immune activation markers	Phase I, active enrollment
Fiber and Probiotic Observational Study (no NCT)	Self-reported fiber and probiotic intake	Melanoma patients on ICIs	ICI response, PFS, microbiome modulation	Observational: probiotics may impair ICI efficacy

**Table 4:** Translational strategies linking diet, metabolism, and melanoma immunotherapy.

Strategy/Focus Area	Mechanism/Rationale	Clinical Application/Potential Impact
Precision Nutrition	Tailors diet to tumor metabolic and immune profiles using metabolomics/microbiome data	Enhances ICI response, reduces resistance, supports personalized treatment plans
Metabolic Profiling	Identifies tumor fuel dependencies (e.g., glucose, amino acids, lipids)	Guides diet-based interventions (e.g., ketogenic, methionine-restricted diets)
Microbiome Modulation	Alters gut flora through fiber-rich diets, prebiotics, or fecal transplants	Increases ICI efficacy; improves T-cell infiltration and cytokine signalingδ
Dietary Pattern Interventions	Uses evidence-based diets (e.g., Mediterranean, FMDs) to reduce inflammation and support immunity	Demonstrates improved ORR and PFS in ongoing clinical trials
Nutrient-Specific Modulation	Targets metabolic pathways (e.g., glutamine, methionine, fructose)	Sensitizes tumors to ICIs, reduces immune suppression in the TME
Non-Invasive Biomarkers	Uses FDG-PET/CT, hyperpolarized 13C-pyruvate imaging, serum lactate, etc.	Monitors metabolic shifts and early therapy response
Sex and BMI Stratification	Accounts for sex-specific metabolism and obesity-related immune modulation	Explains variability in response; supports ‘obesity paradox’ considerations in male patients
Diet-Adherence Technologies	Implements mobile apps, digital food logs, and wearable tech	Improves compliance with complex diets and provides real-time feedback
Patient-Centered Design	Aligns nutrition strategies with patient preferences, barriers, and quality of life goals	Increases feasibility, adherence, and clinical impact
